# Selective targeting of human colon cancer stem-like cells by the mTOR inhibitor Torin-1

**DOI:** 10.18632/oncotarget.1310

**Published:** 2013-09-19

**Authors:** Maria Giovanna Francipane, Eric Lagasse

**Affiliations:** ^1^ McGowan Institute for Regenerative Medicine, Department of Pathology, University of Pittsburgh School of Medicine, Pittsburgh, PA, USA; ^2^ RiMed Foundation, Palermo, Italy

**Keywords:** colorectal cancer, cancer stem cells, mTOR, apoptosis

## Abstract

Metastatic colorectal cancer (CRC) is incurable for most patients. Since mammalian target of rapamycin (mTOR) has been suggested as a crucial modulator of tumor biology, we aimed at evaluating the effectiveness of mTOR targeting for CRC therapy. To this purpose, we analyzed mTOR expression and the effect of mTOR inhibition in cancer stem-like cells isolated from three human metastatic CRCs (CoCSCs).

CoCSCs exhibited a strong mTOR complex 2 (mTORC2) expression, and a rare expression of mTOR complex 1 (mTORC1). This latter correlated with differentiation, being expressed in CoCSC-derived xenografts. We indicate Serum/glucocorticoid-regulated kinase 1 (SGK1) as the possible main mTORC2 effector in CoCSCs, as highlighted by the negative effect on cancer properties following its knockdown. mTOR inhibitors affected CoCSCs differently, resulting in proliferation, autophagy as well as apoptosis induction. The apoptosis-inducing mTOR inhibitor Torin-1 hindered growth, motility, invasion, and survival of CoCSCs *in vitro*, and suppressed tumor growth *in vivo* with a concomitant reduction in vessel formation. Torin-1 also affected the expression of markers for cell proliferation, angio-/lympho-genesis, and stemness *in vivo*, including Ki67, DLL1, DLL4, Notch, Lgr5, and CD44. Importantly, Torin-1 did not affect the survival of normal colon stem cells *in vivo*, suggesting its selectivity towards cancer cells. Thus, we propose Torin-1 as a powerful drug candidate for metastatic CRC therapy.

## INTRODUCTION

Colorectal cancer (CRC) is the second leading cause of cancer death in the United States [1]. Despite new treatment options developed in the last decade, the prognosis for patients with advanced or recurrent CRC remains poor.

The serine/threonine kinase mammalian target of rapamycin (mTOR) has been suggested as a crucial modulator of tumor cell growth and proliferation, and therefore a potential target for anticancer therapy [2]. Unfortunately, drug development against mTOR started when knowledge of its function was very preliminary, resulting in contradictory data and unsuccessful clinical trials. Several breakthroughs have recently changed the course of mTOR-oriented drug discovery. First, the fact that mTOR exists in two distinct complexes: mTORC1 containing Raptor, and mTORC2 containing Rictor [3]. Second, the discovery that Rapamycin (the first mTOR inhibitor identified) exerts an incomplete inhibition of mTORC1 and is inactive against mTORC2 under short-term conditions [4]. Third, the existence of negative feedback loops linking mTOR to other pathways. Akt can phosphorylate mTOR in mTORC1, consequently leading to phosphorylation of ribosomal S6 protein kinase 1 (S6K1) and eIF4E-binding protein 1 (4E-BP1), mediators of protein translation and cell growth [5]. mTORC1-activated S6K1 phosphorylates Rictor and/or Insulin receptor substrate (IRS)-1, thus inhibiting mTORC2 and phosphatidyl inositol 3-kinase (PI3K)/Akt signaling, respectively [6, 7]. More recent findings indicate that mTORC1 also phosphorylates Growth Factor Receptor Bound Protein 10 (Grb10), leading to accumulation of Grb10 and negative feedback inhibition of PI3K and Microtubule-associated protein kinase/Extracellular-signal regulated kinase (MAPK/ERK) pathway [8]. mTORC2 plays an important role in cell survival, metabolism, proliferation and cytoskeleton organization, as it phosphorylates Protein Kinase Cα (PKCα), SGK1, as well as Akt, allowing for its complete activation [3, 9-11]. Documented pro-survival rather than anticancer effects of Rapamycin likely resulted from disruption of the mTORC1-dependent negative feedback loop to mTORC2 and IRS-1/PI3K. This awareness pushed for development of a new generation of inhibitors, which compete with ATP in the catalytic site of mTOR, and inhibit both complexes [12]. Unfortunately, the effects of the so-called mTOR kinase inhibitors (mTorKIs) have been poorly investigated.

Studies in *Apc*Δ716 mice, a mouse model of familial adenomatous polyposis, sustained the basis of mTORC1-targeted drug development for therapy and prevention of colon polyps and cancers [13, 14]. However, blocking a specific pathway may disrupt the balance between signaling pathways and enhance oncogenic signals. In that regard, in parallel with its cytostatic effect, Rapamycin strongly increased MAPK kinase (MEK)/ERK activity, resulting in the appearance of a spindle morphology and higher invasiveness of K-Ras-transformed intestinal epithelial cells (IECs) [15]. This indicated the need for new drugs able to overcome the relief of feedback inhibition of pro-survival, pro-invasive and pro-metastatic pathways. Besides mTORC1, mTORC2 is overexpressed in CRC and plays an important role in cancer biology [16]. Recent studies have demonstrated the efficacy of the dual kinase inhibitors NVP-BEZ235 and pp242 in CRC cell line-derived xenografts [17, 18]. Nevertheless, a remarkable intrinsic resistance of a large proportion of CRC cell lines to mTorKIs, including the above-mentioned compounds, was also described, warranting further studies [19].

With the formulation of the cancer stem cell (CSC) hypothesis in tumors including CRC, it has become clear that successful tumor eradication requires the depletion of a population of precursor/progenitor cells with indefinite self-renewal capacity, chemotherapy resistance, and metastatic ability [20, 21]. Despite this, to our knowledge, only one study investigated the effects of mTOR inhibitors in cancer stem-like cells so far. mTOR signaling was shown to be activated in colorectal cell line-derived spheres in serum-free medium [22]. Treatment with Rapamycin and pp242 diminished sphere-forming capacity as well as ALDH1 activity. However, only pp242 suppressed the enrichment of ALDH^+^ cells induced by chemotherapy, thus highlighting an essential role of the mTORC2 signaling in the maintenance of the CRC stem-like phenotype. Although this study further confirmed the importance of mTOR signaling in CRC, the authors did not perform sufficient functional experiments to assess the effect of mTOR inhibition on biological properties and tumorigenic potentials of CRC stem-like cells. Moreover, the existence of a controversy in the literature as to whether ALDH^+^ cells isolated from cancer cell lines can serve as *in vitro* model for CSC study, further indicates the need to study the effect of mTOR inhibition using alternative methods to identify and characterize CSCs. Multiple cell-surface proteins have been proposed as potential candidate markers for colon stem-like cells (CoCSCs), and our *in vitro* system efficiently enriches for these cells [23]. Here, we first analyzed CoCSCs for expression of major mTORC1/2 pathway components. We then tested different mTOR inhibitors, either alone or in combination with standard chemotherapy. Through these studies, we identified Torin-1 as the most powerful inhibitor among those examined for CRC therapy.

## RESULTS

### mTORC2 likely regulates physiology of both colon cancer progenitor and mature cells, while mTORC1 likely contributes to CoCSC differentiation

**Figure 1:**
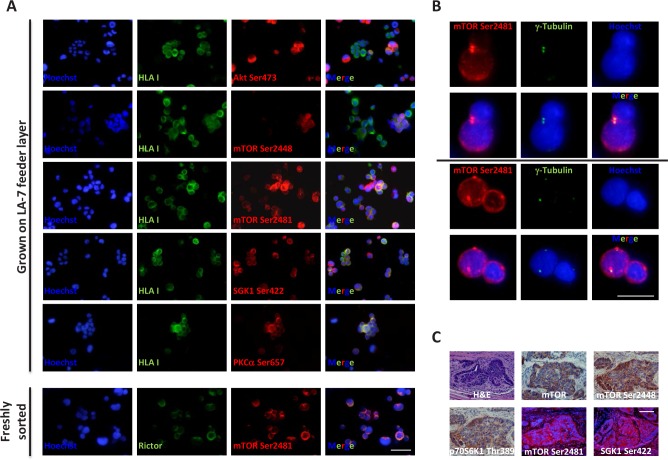
Expression of mTORC1/2 pathway components in CoCSC-derived cultures and xenografts Immunofluorescence pictures of Tu22 showing (A) expression of distinct mTOR pathway components, and (B) co-localization of mTOR Ser2481 and γ-tubulin. Scale bars, 100μm. (C) Hematoxylin and eosin (H&E), immunohistochemistry (AEC, red color) or immunofluorescence staining for different mTOR pathway components on PFA-fixed, paraffin-embedded serial sections of Tu12-derived xenografts. Scale bar, 100μm.

Although several mTOR pathway components have been investigated in a number of cancers including those of the colon [24], to our knowledge, no study investigating their expression in patient-derived CoCSCs has been reported so far. By immunofluorescence, we therefore analyzed the expression of Akt Ser473, mTOR Ser2448, mTOR Ser2481, SGK1 Ser422, and PKCα Ser657, in CoCSCs derived from three human metastatic CRCs (Tu12, Tu21, and Tu22 cells) [23]. Since these cells were grown on a rodent feeder layer, co-staining with an anti-HLA antibody was necessary to discriminate human (CRC) *versus* non-human (stroma) cells. Comparable results were obtained in all three cell lines tested. CoCSCs exhibited unexpectedly low Akt signaling but mTORC2 activation, as revealed by strong phosphorylation in all the cells of mTOR at Ser2481 and of its effectors SGK1 and PKCα, at residues previously reported to be modified following mTORC2 activation (Figure 1A) [2]. A rare positivity for mTOR Ser2448 (indicative of mTORC1 activation status [2]) and infrequency of Thr389 phosphorylation of the p70S6K1 mTORC1 effector (*data not shown*) were also observed, indicating that as compared to mTORC1, mTORC2 might play a much more important role in regulating CoCSC physiology. Moreover, mTOR Ser2481 and Rictor co-localized, further indicating mTORC2 activation in our system. Interestingly, mTOR Ser2481 co-localized with γ-tubulin in centrosomes, thus suggesting a potential role for mTORC2 in the control of CoCSC proliferation (Figure 1B). While mTOR Ser2448 and p70S6K1 Thr389 were barely detectable *in vitro*, they were clearly expressed *in vivo*, together with mTOR Ser2481 and SGK1 Ser422 (Figure 1C). Because CoCSC differentiation has been previously observed after transplantation in immunodeficient mice [23], our immunostaining suggests a possible regulatory role of mTORC1 in CoCSC differentiation and of mTORC2 in the physiology of both stem/progenitor and mature tumor cells.

### SGK1 facilitates CoCSC growth and drug resistance

**Figure 2:**
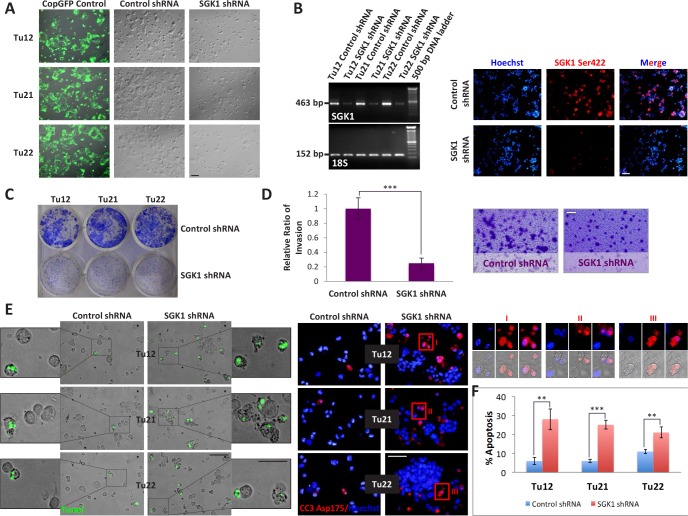
SGK1 knockdown reduces CoCSC growth, invasive ability and chemoresistance Tu12, Tu21 and Tu22 were infected with copGFP control, control shRNA or SGK1 shRNA lentiviral particles (MOI=6.5). 72h post-transfection, cells were subjected to (A) light microscopy, and (B, *left*) RT-PCR analysis for SGK1 or 18S housekeeping gene as internal control. Scale bars, 100μm. After additional 5 days of puromycin selection (1.5-2μg/mL), cells were analyzed by immunofluorescence for SGK1 Ser422 (B, *right*). Scale bar, 100μm. (C) Decreased CoCSC clonogenicity following SGK1 knockdown. One hundred thousand cells infected with control or SGK1 shRNA lentiviral particles were plated in each well of a 6-well plate. 2 weeks later, colonies were fixed and stained with 0.2% crystal violet (CV) in 10% ethanol. (D) Bar graph (*left*) and optical imaging (*right*) of CV-stained transwells showing reduced invasive potential of SGK1-silenced Tu12 cells. Scale bar, 100μm. Fifteen thousand cells infected as in (C) were seeded on growth factor reduced Matrigel (2mg/ml)-coated transwells and allowed to invade for 48h. Data are presented as relative ratio of invasion from one experiment representative of two independent experiments carried out in triplicate (***p<0.001) (the regions representing invaded cells were selected using the “Magic Wand” tool, and the highlighted pixels counted using the histogram command in Adobe Photoshop). (E) Merged fluorescent TUNEL assay and optical imaging (*left*; scale bars, 100 or 50μm), and cleaved caspase-3 (CC3) Asp175 immunofluorescence (*right*, scale bar, 100μm) of Tu12, Tu21 and Tu22 cells infected as in (C) following 1μM Oxaliplatin treatment for 48h. Enlarged images (I, II, III) show CC3 reactivity in apoptotic bodies. (F) Bar graph showing mean percentage (±SD) of apoptotic cells calculated considering three different microscopic fields of TUNEL assay and CC3 staining (**p<0.01, ***p<0.001).

The finding that CoCSCs expressed low levels of activated Akt might be explained in light of the recent observation that activated Akt in CRC correlates with low stage and good prognosis, [25] and this was not our case, since we were analyzing cells isolated from CRC metastases to the liver [23]. The fact that despite low Akt activation, CoCSCs showed mTORC2 activation, led us to hypothesize that Akt could not have been a major mTORC2 effector in our system. SGK1 is the main mTORC2 effector in yeast and worms [26]; thus, we hypothesized that this could also have been the case with CoCSCs. To prove this hypothesis, we investigated whether SGK1 knockdown could affect cancer properties. Tu12, Tu21 and Tu22 cells were purified from contaminating feeder cells, grown on plastic, and infected with copGFP control, control shRNA or SGK1 shRNA lentiviral particles (Figure 2A). RT-PCR analysis revealed a significant decrease in SGK1 mRNA levels 72h after transfection (Figure 2B, *left*), while immunofluorescence analysis highlighted a strong decrease of SGK1 phosphorylation following puromycin selection (Figure 2B, *right*). Decreased clonogenicity (Figure 2C), invasive ability (p=0.0006) (Figure 2D), and a 2-5fold increased Oxaliplatin-induced apoptosis (Figure 2E–F), were also observed following SGK1 knockdown. Thus, rather than Akt, SGK1 could be the main mTORC2 effector in CoCSCs.

### mTOR inhibitors differently affect CoCSC viability

**Figure 3:**
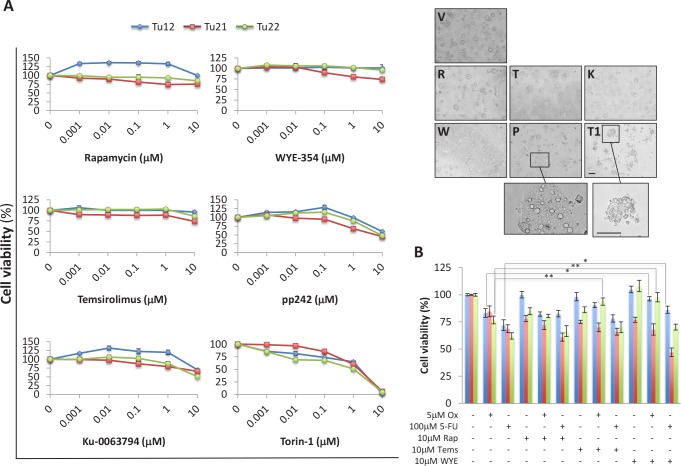
Differential effects of mTOR inhibitors on CoCSC viability (A) Line graphs (*left*) and optical imaging (*right*) showing viability of Tu12, Tu21 and Tu22 cells treated with vehicle (V) or a serial dilution (0.001-10μM) of Rapamycin (R), WYE-354 (W), Temsirolimus (T), pp242 (P), Ku-0063794 (K) or Torin-1 (T1). Scale bars, 200μm. The number of viable cells was measured by MTS assay after 72h of drug exposure. Data are expressed as mean percentage (±SD) of viable cells as compared to respective control cultures, obtained from triplicate absorbance readings from at least three independent experiments. Optical images were taken from Tu22 cells exposed to 10μM of each drug for 72h. (B) Bar graph showing mean percentage (±SD) of viable cells following treatment with Oxaliplatin (Ox, 5μM) or 5-Fluorouracil (5-FU, 100μM) alone or in combination with 10μM Rapamycin (Rap), Temsirolimus (Tems) or WYE-354 (WYE) for 72h. The number of viable cells was obtained as in (A) (*p<0.05, **p<0.01).

Since mTORC2 activation characterized CoCSCs *in vitro*, we compared the effects of various mTorKIs (Ku-0063794, WYE-354, pp242, and Torin-1) to first-generation mTOR inhibitors (Rapamycin and Temsirolimus) on the three CoCSC lines previously examined. Feeder cell-depleted CoCSCs were treated with vehicle or a serial dilution (0.001-10μM) of the above-mentioned compounds for 72h. Even high drug concentrations were often not sufficient to affect cell viability (Figure 3A, *left*). Interestingly, mTOR inhibitors sometimes induced cell proliferation. Only Torin-1 decreased cell viability in a dose-dependent fashion. 1μM decreased Tu12, Tu21, and Tu22 cell viability to 65% ± 2.3, 60% ± 5.2, and 51% ± 4.3%, respectively, while 10μM killed all of the cells. 10μM pp242 caused the accumulation of large cytoplasmic vacuoles inside the cells (Figure 3A, *right*). Additional studies revealed that pp242 induced autophagy, as indicated by acidic vesicular organelle (AVO) formation, changes in the localization of the autophagosome marker LC3 from diffuse cytosolic to a punctate distribution (Supplementary Figure 1A), and increased Beclin-1 mRNA levels (Supplementary Figure 1B). A late-stage inhibitor of autophagolysosome formation, Bafilomycin A1, effectively blocked the accumulation of pp242-induced AVOs, but did not further sensitize cells to the effects of mTOR inhibition (Supplementary Figure 1C). In contrast, autophagy inhibition alleviated pp242 cytostatic effects, as proliferative rates of treated cells returned to basal levels (*data not shown*). Moreover, a transient and weak S phase cell cycle arrest accompanied pp242-induced autophagy (Supplementary Figure 1D), further indicating that autophagy was not delaying apoptotic cell death, but effectively rescuing cells from death.

As expected, both Rapamycin and Temsirolimus did not affect mTOR Ser2481 phosphorylation (Supplementary Figure 2A) and low Rapamycin doses (10nM) induced Akt feedback activation (Supplementary Figure 2B). p70S6K1 Thr389 levels were very low in CoCSCs and not altered by Rapamycin exposure up to 1μM. Conversely, Rapamycin markedly decreased Grb10 protein abundance (Supplementary Figure 2C), suggesting that the relief of Grb10-mediated negative feedback inhibition of PI3K may have sometimes induced CoCSCs to activate Akt and proliferate.

We then aimed at testing mTOR inhibitors in combination with standard chemotherapy. Tu12, Tu21 and Tu22 cells were treated with 5μM Oxaliplatin or 100μM 5-Fluorouracil alone or in combination with 10μM Rapamycin, Temsirolimus or WYE-354 for 72h. The mTOR inhibitors tested did not show additive or synergistic effects in combination with chemotherapeutics (Figure 3B). Rather, Temsirolimus rescued Oxaliplatin-treated Tu22 cells (p=0.0075), while WYE-354 rescued Oxaliplatin-treated Tu12 (p=0.04) and Tu22 cells (p=0.008), as well as 5-Fluorouracil-treated Tu12 cells (p=0.03). Thus, mTOR inhibitors may have a beneficial role by protecting cells against pro-apoptotic insults. There was no observed benefit of combining Ku-0063794, Torin-1 or pp242 with chemotherapeutics (*data not shown*). Taken together, Torin-1 as a single agent, is the most powerful inhibitor among those examined for CRC therapy, as it efficiently suppresses tumor growth *in vitro*.

### Torin-1 triggers CoCSC apoptosis

**Figure 4:**
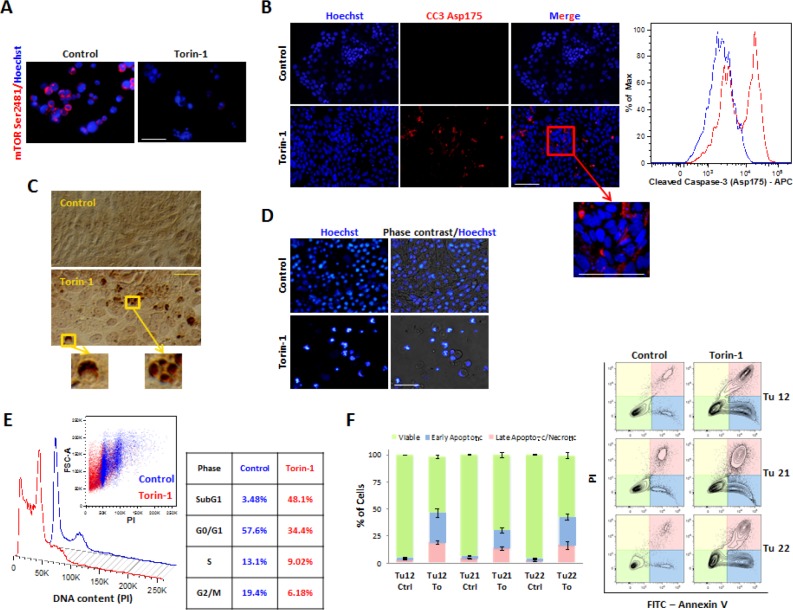
Torin-1 triggers CoCSC apoptosis Immunofluorescence pictures of control or Torin-1-treated (5μM, 15 h) Tu12 cells showing expression of (A) mTOR Ser2481 and (B, *left*) cleaved caspase-3 (CC3) Asp175. Scale bars, 100μm. (B, *right*) Flow cytometry histogram showing CC3 Asp175 staining on PFA- and methanol-fixed Tu12 cells treated as in (A). (C) Optical imaging of TUNEL assay on Tu12 cells treated as in (A) (DAB, brown color). Scale bar, 100μm. (D) Hoechst 33342 staining and merged pictures of Hoechst staining and optical imaging of Tu12 cells treated with 5μM Torin-1 or vehicle as control for 72h. Scale bar, 100μm. Condensed chromatin, fragmented nuclei, and apoptotic bodies can be observed following treatment. (E) DNA content histogram (*left*) and table (*right*) showing sub-G1 cell accumulation after Torin-1 treatment (5μM, 15h). Tu12 cells were fixed with 70% ethanol, RNase treated (0.2mg/ml), and stained with PI (40μg/ml) before being analyzed with a flow cytometer. Quantitative analysis is based on the Dean-Jett-Fox curve-fitting model. (F, *left*) Stacked bar graph showing mean percentage (±SD) of viable, early apoptotic or late apoptotic/necrotic Tu12, Tu21 or Tu22 cells treated as in (A), determined using FITC Annexin V/PI staining. Results are representative of at least two experiments. (F, *right*) Flow cytometry contour plots of FITC Annexin V/PI-stained Tu12, Tu21 and Tu22 cells treated as in (A).

Various techniques were used to determine whether Torin-1 induced apoptosis. 5μM Torin-1 treatment for 15h suppressed mTOR Ser2481 phosphorylation (Figure 4A) and induced apoptosis, as indicated by caspase-3 activation, monitored by both immunofluorescence and flow cytometry analysis (Figure 4B), and appearance of TUNEL-positive half moon shaped and fragmented nuclei (Figure 4C). Following 72h of treatment, all the cells were apoptotic (Figure 4D). Importantly, 5μM Torin-1 treatment for 15h resulted in sub-G1 cell accumulation (Figure 4E), and appearance of single Annexin V^+^ cells (Figure 4F), both indicative of apoptosis induction.

Emergence of resistant clones represents a main problem of current therapies. To understand whether CoCSCs could develop Torin-1 resistance, Torin-1 resistant Tu12, Tu21, and Tu22 cells were generated, as depicted in Supplementary Figure 3A. Cells could now survive 5μM Torin-1, but they were no longer able to extensively proliferate *in vitro* (Supplementary Figure 3B). S.c. injection of Torin-1 resistant cells into mice (n=7) did not generate palpable tumors during a 7-wk observation period (Supplementary Figure 3C). Nevertheless, examination of skinned mice revealed two mice had formed very small tumors. Thus, CoCSC cultures that have been subjected to a prolonged, continuous, multistep selection with Torin-1 contain a strikingly reduced tumor-initiating cell population, thus encouraging Torin-1 potential use for CRC therapy.

### Torin-1 hinders growth, motility, invasion, and survival of distinct CoCSC subpopulations

**Figure 5:**
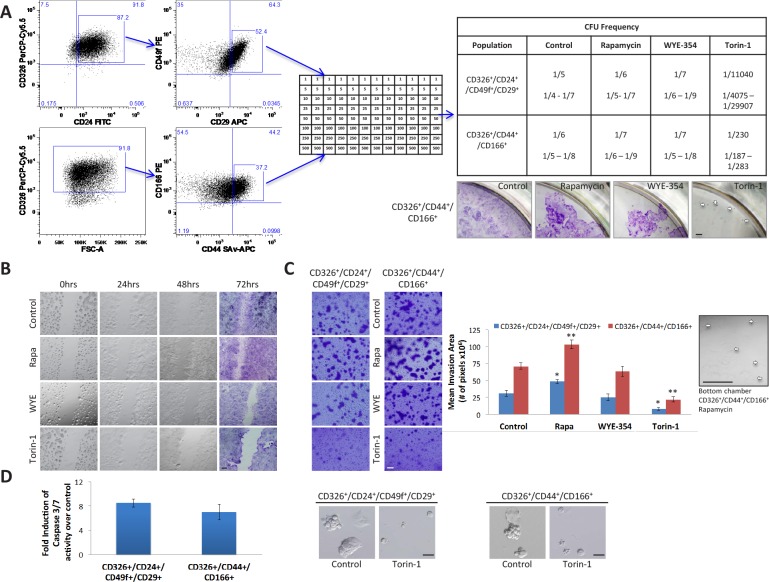
Rapamycin, WYE-354 and Torin-1 differently affect cancer properties of distinct CoCSC subpopulations (A) Flow cytometry dot plots (*left*) showing gates used for sorting Tu12 cells. Cells were stained with CD326 PerCP-Cy5.5, CD24 FITC, CD49f PE, CD29 APC, and Sytox Blue or CD326 PerCP-Cy5.5, CD166 PE, CD44 SAv-APC, and Sytox Blue. CD326^+^/CD24^+^/CD49f^+^/CD29^+^ and CD326^+^/CD44^+^/CD166^+^ subpopulations were sorted directly into 96-well plates in medium containing vehicle, 1μM Rapamycin, WYE-354 or Torin-1 (see the table for cell densities, *middle panel*). Medium was replaced every 72h up to 12 days, when wells containing colonies were scored and CFU frequencies (±SE) estimated using the L-Calc™ software (StemCell Technologies) (*upper right panel*). Optical imaging of CV-stained wells (*lower right panel*) showing confluence rates of CD326^+^/CD44^+^/CD166^+^ cells exposed to different drugs (pictures are from wells originally plated with the highest number of cells; white arrows indicate colonies). Scale bar, 200μm. (B) Optical imaging of unstained or CV-stained CD326^+^/CD44^+^/CD166^+^ cells treated as in (A) and migrating across the gap up to 72h. Scale bar, 200μm. Thirty thousand cells were seeded into each well of a culture insert and grown overnight. After removal of the insert, a 500μm cell-free gap was created. Cells were therefore treated, and migration monitored at different time points. (C) Optical imaging of CV-stained transwells (*left*) showing invasive potential of CD326^+^/CD24^+^/CD49f^+^/CD29^+^ and CD326^+^/CD44^+^/CD166^+^ Tu12 cells treated as in (A). Scale bar, 100μm. Fifteen thousand cells were sorted directly into growth factor reduced Matrigel (2mg/ml)-coated transwells and allowed to invade for 72h. Drugs were added to bottom chambers. Bar graph (*middle*) shows the mean (±SD) invasion area (pixels) from one representative experiment of two in triplicate, with similar results (statistical analysis was performed with respect to control samples; *p<0.05, **p<0.01). Optical imaging (*right*) of a bottom chamber showing CD326^+^/CD44^+^/CD166^+^ cells crossing the porous membrane under Rapamycin exposure (white arrows indicate cells). Scale bar, 100μm. (D) Bar graph (*left*) showing relative caspase 3/7 activity levels (fold increase over control) of CD326^+^/CD24^+^/CD49f^+^/CD29^+^ and CD326^+^/CD44^+^/CD166^+^ Tu12 cells following treatment with vehicle or Torin-1 (5μM, 15h), and optical imaging (*right*) showing morphological changes in treated *versus* control cells. Scale bars, 200μm. Data of caspase 3/7 activities are presented as mean (±SD) of the luminescence values obtained in triplicate determination from at least three independent experiments.

Despite the first wave of enthusiasm surrounding the CSC field, no consensus has emerged so far about cell surface marker profiles that define CoCSCs, Initially described as a unique marker for immature intestinal cells, CD133 was later subject of huge controversy [27]. Conversely, the combined expression of CD326^high^/CD44^+^/CD166^+^ was suggested as being more robust for CoCSC isolation [28]. Both CD24^+^/CD29^+^ and CD24^+^/CD49f^+^ signature have been suggested to characterize putative mammary stem/progenitor cells [29]. Interestingly, we found colony-forming unit (CFU) frequencies of CD326^+^/CD24^+^/CD49f^+^/CD29^+^ and CD326^+^/CD44^+^/CD166^+^ CRC subpopulations to be very similar. For this reason, we chose these two subpopulations within Tu12 cells to further confirm Torin-1 anti-CoCSC activity. Particularly, we performed limiting dilution analysis, migration, and invasion assays, in the presence or absence of 1μM Rapamycin, WYE-354, or Torin-1. While CFU frequencies among Control, Rapamycin-, and WYE-354-treated cells were similar, CFU frequencies following Torin-1 treatment were significantly decreased (Figure 5A, *upper right panel*). However, cells exposed to Rapamycin or WYE-354 were less confluent than control cells, and Torin-1-treated cells generated very small colonies (Figure 5A, *lower right panel*). Compared to control cells, more cells migrated through the gap at 48h and invaded the Matrigel layer at 72h under Rapamycin exposure (p=0.035 for CD326^+^/CD24^+^/CD49f^+^/CD29^+^, and 0.0056 for CD326^+^/CD44^+^/CD166^+^ cells) (Figure 5B–C). Moreover, Rapamycin caused some cells to cross the porous membrane and adhere to the bottom chamber (Figure 5C, *right*). In contrast, control cell were completely unable to cross the membrane under experimental conditions. Although 72h WYE-354 treatment did not affect Tu12 cell viability (Figure 3A, *left*), it slightly reduced migration, but not invasion of both subpopulations (p=0.37 and p=0.36, respectively) (Figure 5B–C). Conversely, Torin-1 strongly inhibited both migration and invasion of the selected subpopulations (p=0.024 and 0.009, respectively), and activated apoptosis as evidenced by increased caspase 3/7 activity and morphological changes (Figure 5B–D), confirming its anti-CSC activity.

### Torin-1 reduces tumor growth and vessel formation *in vivo*

**Figure 6:**
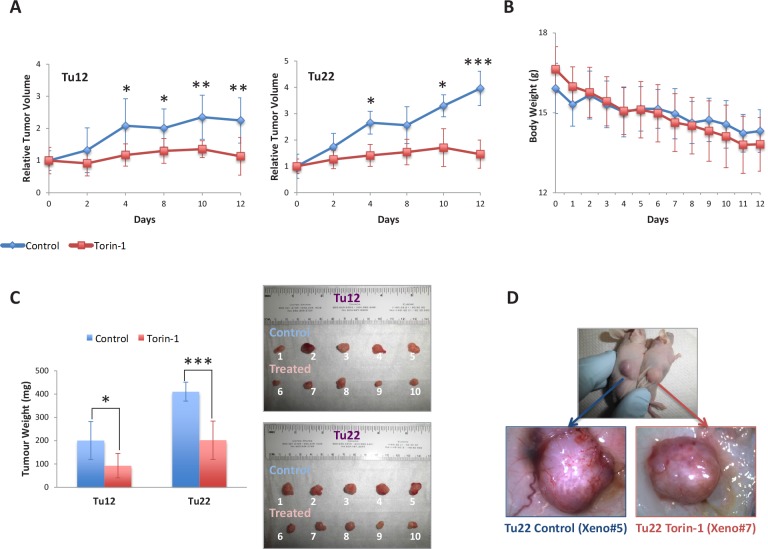
Torin-1 suppresses CRC growth and angiogenesis (A) Line graphs showing relative Tu12 (*left*) or Tu22 (*right*) tumor volumes (ratios of tumor volumes to initial size at start of treatment) for control and treated mice as a function of time. 20mg/Kg Torin-1, or vehicle was delivered by i.p. injection once daily for 12 days after the tumor reached the size of 150-250 mm^3^ (19 days after transplantation of 5×10^5^ tumor cells/mouse; statistical analyses were performed with respect to control tumors; *p<0.05, **p<0.01, ***p<0.001). (B) Line graph showing control and treated mouse body weight (g) over a 12-day period. (C, *left*) Bar graph showing the weight (mg) of Tu12 and Tu22 xenografts excised from both control and treated groups (*p<0.05, ***p<0.001). (C, *right*) Macroscopic appearance of all xenografts excised. (D) Tu22 tumor–bearing mice after 12 days treatment with vehicle (*upper picture, left mouse*) or Torin-1 (*upper picture, right mouse*), and stereoscopic images of respective excised tumors (Xeno#5 and Xeno#7, *lower pictures*) showing different vascularization.

To test whether Torin-1 could suppress CRC progression *in vivo*, we established Tu12 and Tu22 xenografts in nude mice. Then, mice received 20mg/Kg Torin-1 or vehicle every day up to 12 days. Torin-1-treated tumors grew slower than control tumors (Figure 6A). Drug was well tolerated as no significant differences in body weight between the two groups were observed (Figure 6B). The mean weights of the excised tumors were approximately 50% less in mice treated with Torin-1 with respect to control mice (Tu12, p=0.025; Tu22, p=0.0009) (Figure 6C). Importantly, tumor vasculature was less developed in treated tumors (Figure 6D), suggesting Torin-1 anti-angiogenic activity.

### Torin-1 decreases the expression of proliferative, angio-/lympho-genic, and stem cell markers, and activates apoptosis *in vivo*

**Figure 7:**
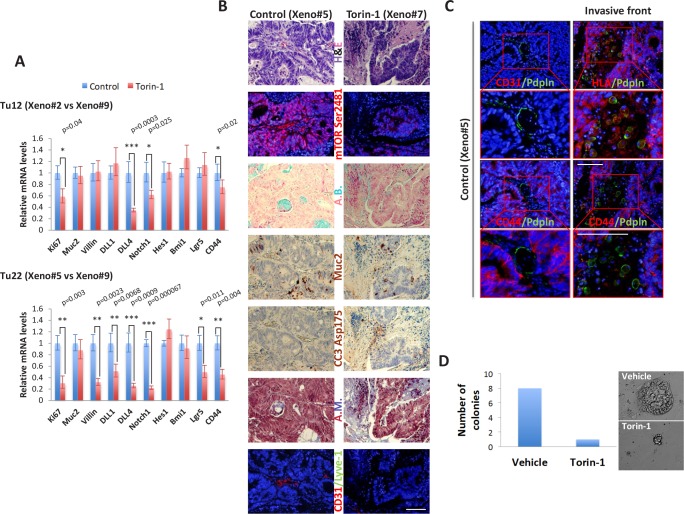
Torin-1 decreases the expression of proliferative, angio-/lympho-genic, and stem cell markers, and activates apoptosis *in vivo* (A) Bar graphs showing relative real-time qPCR analysis of Ki67, Muc2, Villin, DLL1, DLL4, Notch1, Hes1, Bmi1, Lgr5, and CD44 in control or Torin-1-treated Tu12 (Xeno#2 *vs* Xeno#9, *upper*) or Tu22 (Xeno#5 *vs* Xeno#9, *lower*) tumors. Expression was normalized to *GAPDH* mRNA. Error bars represent upper and lower error limits based on replicate variability (*p<0.05, **p<0.01, ***p<0.001). (B) Images of PFA-fixed, paraffin-embedded serial sections of control or Torin-1-treated Tu22 tumors (Xeno#5 *vs* Xeno#7) stained with H&E, mTOR Ser2481, Alcian Blue (A.B.), Muc2 (AEC, red color), cleaved caspase-3 (CC3) Asp175 (AEC, red color), Azan Mallory (A.M.), or CD31/Lyve-1. Scale bar, 100μm. (C) Immunofluorescence pictures of control Tu22 tumor (Xeno#5) sections showing Podoplanin (Pdpln) expression in both lymphatic vessels (*left*) and tumor cells (*right*) at the invasive front. Scale bars, 100μm. Podoplanin^+^ vessels were CD31^−^. The human origin of Podoplanin^+^ cells located outside vessels was confirmed by HLA staining. Podoplanin^+^ cells exhibited round morphology and were CD44^−^. (D) Bar graphs showing number of colonies generated from CD326^+^ cells isolated from vehicle or Torin-1-treated xenografts. 10^3^ cells were plated on a 24-well plate previously coated with LA7 feeder layer cells, and grown for 1 week before scoring the number of colonies.

Different markers were evaluated by real-time qPCR analysis in both untreated and treated tumors (Figure 7A). With respect to control tumors, the proliferation marker Ki67, the Notch pathway members involved in angiogenesis DLL4 and Notch1 [30], and the CSC surface marker CD44 were significantly reduced in both Tu12 and Tu22 treated tumors. No changes in the mRNA levels of the goblet cell differentiation marker Muc2, the Notch effector Hes1, and the Polycomb member Bmi1 were observed. The enterocyte differentiation marker Villin, the Notch pathway component DLL1, and the crypt stem cell marker Lgr5 were decreased in Tu22, but not in Tu12 tumors, following treatment. Anti-CSC therapy is expected to promote differentiation; the reduction in Villin levels may therefore seem paradoxical. However, a previously unrecognized function of Villin in IEC survival was reported [31], indicating that Torin-1 might favor apoptosis by reducing Villin levels. A decrease in mTOR Ser2481, and an increase in CC3 and fibrotic tissue (Azan Mallory, A.M.) were observed in treated *versus* control tumors (Figure 7B). In accordance with molecular analysis, no changes in goblet cell numbers were found, as investigated by Muc2 and Alcian Blue (A.B.) stainings (Figure 7B). Importantly, treated tumors contained fewer blood vessels, as examined through CD31 staining (Figure 7B). Interestingly, Podoplanin expression characterized both lymphatic vessels and tumor cells at the invasive front of control tumors, while no positivity was observed in treated tumors (Figure 7C). Podoplanin^+^ vessels were CD31^−^. Podoplanin^+^ cells located outside vessels were human in origin, although HLA expression was dispersed throughout their cytoplasm. This is not surprising since tumor cells often down-regulate HLA antigens surface expression to escape immunological attack. Podoplanin^+^ cells exhibited round morphology typical of amoeboid motility and were CD44^−^. Loss of CD44 expression in invaded area is a good indicator of lymph-node metastasis in CRC [32]. Thus, while control tumors comprised cells with high metastatic potential, cells in treated tumors were less prone to migrate to distant sites.

Since *in vitro* cultured tumor cells might not maintain the exact properties of the primary tumor, by acquisition of additional gene alterations, we aimed at assessing Torin-1 effects on xenografts obtained from injection of freshly isolated metastatic CRC cells. After 5 months from injection, 6 out of 10 tumors had formed. Mice were therefore divided in two groups, and treated with 20mg/Kg Torin-1 or vehicle every day up to 12 days (parental tumor was confirmed for mTOR pathway activation before beginning Torin-1 treatment, *data not shown*). Torin-1-treated tumors grew slower than control tumors (*data not shown*). Importantly, CD326 expression was reduced after treatment, as assessed by flow cytometry analysis on freshly isolated cells (*data not shown*). CD326 is one of the first and prominent immunotherapeutic targets in cancer therapy due to its frequent and high-level expression on most carcinomas of various origins. It can abrogate E-cadherin mediated cell-cell adhesion thereby promoting metastasis [33], and can support proliferation by enhancing Wnt signaling [34]. CD326^+^ cells from both groups were sorted and plated on stroma feeder layer. CD326^+^ cells isolated from vehicle-treated xenografts generated a higher number of colonies than CD326^+^ cells isolated from Torin-1-treated xenografts, and colonies were significantly bigger (Figure 7D). All these data support the potential of further development of Torin-1 as therapeutic for the treatment of CRC.

### Torin-1 does not affect the survival of normal colon stem cells *in vivo*

**Figure 8:**
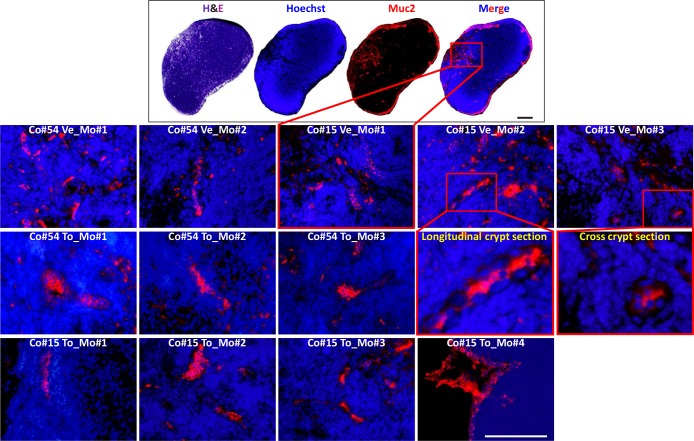
Torin-1 does not affect the survival of normal colon stem cells in vivo (*upper panel*) H&E or immunofluorescence staining of PFA-fixed, paraffin-embedded sections of a mouse lymph node showing engraftment of human fetal intestinal epithelial cells as revealed by Muc2 positivity. Scale bar, 100μm. (*lower panel*) Representative Muc-2 reactive-colon crypts repopulating lymph nodes of mice (Mo) receiving injection of human fetal colon #54 or 15 (Co#54 and Co#15, respectively), and subjected to vehicle or Torin-1 treatment (Ve and To, respectively). Scale bar, 100μm. Enlargements of boxed regions for images Co#15 Ve_Mo#2 and Co#15 Ve_Mo#3 are shown.

Because normal stem cells and CSCs share many traits, it seems reasonable to think that any therapy targeting CSCs may also destroy healthy tissues [35]. Given our recent finding that the mouse lymph node can be used as an *in vivo* organ factory to build up complex organ structures [36], we aimed at assessing the *in vivo* toxicity of Torin-1 against normal colon stem cells by recapitulating colon organ inside the mouse lymph node. To this purpose, we used human fetal intestinal epithelial cells, thought to be enriched in normal stem cells (Figure 8, *upper panel*). Following 3 weeks from cell transplantation, mice were treated with vehicle or 20mg/Kg Torin-1 once a day for 12 days. Tridimensional crypts could be observed in lymph nodes of both untreated and treated mice, to the same extent (Figure 8, *lower panel*). Thus, Torin-1 did not affect the survival of normal colon stem cells *in vivo*, suggesting its selectivity towards cancer cells.

## DISCUSSION

Although during the last decades *in vivo* preclinical studies revealed the antitumor effect of several drugs against CRC, most patients experience a tumor recurrence. The main explanation for such a dismal prognosis is that common drugs leave behind CoCSCs, whose drug-induced positive selection renders tumors more aggressive. As cancer's *Achilles' heel*, CSCs have been intensively studied to develop more effective therapies. Because it plays such a crucial role in cancer biology, mTOR has emerged as a potential target for drug development. Several mTOR inhibitors, including Rapamycin, have already gone through clinical trials as single agents for treating various cancers without great success. Of the huge list of mTOR inhibitors developed, only Temsirolimus and Everolimus have been approved by the FDA for the treatment of advanced renal cell carcinoma [37]. Nevertheless, the role of mTOR inhibitors in cancer therapy continues to evolve, as new compounds are synthetized. Despite this, data comparing the effects of distinct mTOR inhibitors on CSCs are incomplete.

Here, we first analyzed metastatic CRC cells with the properties of intestinal stem cells as well as tumor-initiating cells for mTOR activation. We found mTOR complexes to be differently expressed in these cells, concluding that mTORC2 might be involved in the maintenance of the cancer stem-like phenotype, while mTORC1 might be involved in CoCSC maturation. This is in agreement with previous studies showing a role for mTORC1 in driving the differentiation of normal and cancer stem/progenitor cells [38-41].

CoCSCs expressed low levels of Akt Ser473, indicating that this kinase could not have been a major mTORC2 effector in our system. We indicated SGK1 as the possible main mTORC2 effector in CoCSCs, as highlighted by the negative effect on cancer properties following its knockdown. Akt hypophosphorylation and dependence of SGK family members for viability are known to occur most frequently in the context of wild-type Phosphatase and tensin homolog (PTEN), and helical PI3K alpha catalytic subunit kinase domain (PIK3CA) mutations [42]. Future studies will be conducted to confirm whether this genetic signature can predict resistance or sensitivity of CoCSCs to the different mTorKIs here studied. Unfortunately, the prognostic and predictive value of common mutations in patients with colon cancer is controversial, due to bias in research settings [43]. In our opinion, mTorKI resistance might also occur through less well-studied but equally important epigenetic mechanisms [44].

We therefore analyzed the effects of six mTOR inhibitors, including Torin-1, a highly potent and selective mTorKI [45]. Of the examined compounds, Torin-1 proved the most powerful inhibitor, suppressing CoCSC growth both *in vitro* and *in vivo*.

mTOR inhibitors can relieve negative feedbacks, resulting in strengthened oncogenic signals. This happened under Rapamycin exposure, which drove cells towards a pro-metastatic phenotype, confirming the notion that Rapamycin can have pro-tumor effects. Nevertheless, despite its role in activating oncogenetic pathways, Rapamycin has also been considered a tumor-preventive agent since it delays carcinogenesis in tumor-prone p53^+/−^ and p53^−/−^ mice, most likely by slowing down the process of aging [46, 47]. mTOR inhibitors can have a double-edged sword by activating autophagy, and autophagy itself has a double-edged sword in cancer by the promotion of metabolically stressed tumor cell survival. Indeed, pp242 protected CoCSCs from the effects of mTOR inhibition through autophagy activation, warranting considerable attention on newly developed inhibitors. The goal of any therapeutic strategy is to trigger CSC apoptosis, and Torin-1 successfully achieved this aim both *in vitro* and *in vivo*. It is possible that higher Torin-1 concentrations could increase antitumor response *in vivo*. However, 20mg/Kg Torin-1 was sufficient to hinder CoCSC expansion, angio-/lympho-genesis, and invasion *in vivo*. Importantly, Torin-1-resistant CoCSCs exhibited much reduced tumorigenicity or were even non-tumorigenic. Moreover, Torin-1 did not affect the survival of normal colon stem/progenitor cells *in vivo*, thus encouraging its potential use for metastatic CRC therapy. Recently, Torin-2, which has better pharmacokinetic properties and an improved synthetic route than Torin-1 has been synthetized [48]. Although to our knowledge no studies have been reported so far to elucidate its effectiveness in cancer, we do not exclude it could also be used to successfully treat CRC. Inappropriate activation of PI3K signaling is one of the most common features of CRC. Several studies have shown that inhibition at multiple levels of the PI3K pathway results in FOXO-dependent feedback reactivation of several receptor tyrosine kinases (RTKs), which, in turn, limit the sustained inhibition of this pathway [49]. This suggests that if used as single agents, PI3K pathway inhibitors may have limited clinical activity. Conversely, dual inhibition of PI3K and mTORC1/2 signaling induced tumor regression in several *in vivo* human tumor xenograft models, and might show greater efficacy than mTorKIs at depleting CoCSCs [50].

## METHODS

### Cells

CoCSCs were cultured as previously described [23].

### Reagents, plastics, and instruments:

Antibodies used are indicated in Supplementary Table 1. CopGFP control (sc-108084), control shRNA (sc-108080), and SGK1 shRNA (sc-38913-V) lentiviral particles, and SGK1 primers (sc-38913-PR) were purchased from Santa Cruz. Sequences of other primers used are indicated in Supplementary Table 2. Drugs were purchased as follows: Rapamycin (R-5000) from LC Laboratories; WYE-354 (CD0270), pp242 (CD0258), and Ku-0063794 (CD0274) from Chemdea; Temsirolimus (PZ0020), 5-Fluorouracil (F6627), and Oxaliplatin (O9512) from Sigma; Torin-1 (1222998-36-8) from Tocris Biosciences. All drugs except Oxaliplatin were dissolved in DMSO as stock solutions, and then diluted with culture medium. Oxaliplatin was dissolved in deionized water. Kits were purchased as follows: RNeasy Mini Kit (74104) from Qiagen; iScript™ Reverse Transcription Supermix for RT-qPCR (170-8841), iTaq DNA Polymerase kit (170-8870), and SsoAdvanced™ SYBR® Green supermix (172-5261) from Bio-Rad; CellTiter 96® AQueous Assay (G3580), DeadEnd™ Colorimetric TUNEL System (G7360), and Caspase-Glo® 3/7 Assay (G8091) from Promega. Transwells (07-200-150) were purchased from Corning. Ibidi culture inserts (80209) were used for migration assay. Flow analysis was performed on a Miltenyi MACSQuant Analyzer. Sorting was performed on a BectonDickenson FACSAria II SORP cell sorter. Final data analysis was done using FlowJo software (TreeStar). A C1000 thermal cycler (Biorad) and a StepOnePlus™ Real-Time PCR System (Applied Biosystems) were used for gene amplification.

### Colon cancer xenografts and treatment

Tumor tissues were minced into small fragments with scalpels and then digested using a two-step collagenase method. Freshly isolated cells from primary tumors or CoCSCs (5 × 10^5^) were suspended in HBSS:Matrigel (1:1) and injected s.c. into both flanks of 5-week-old BALB/c nude mice (Charles River, n=5 per group). 20mg/Kg Torin-1, as a suspension in 20% N-methyl-2-pyrrolidone/40% PEG400/40% water [45], or vehicle was delivered by i.p. injection once daily for 12 days after the tumor was established. Tumors were measured every day using a caliper and tumor volumes calculated according to the formula V=π/6×(larger diameter)×(smaller diameter)^2^. At the end of the treatment mice were killed by CO_2_ euthanasia. Xenografted tumors were excised, weighed, photographed, fixed in buffered formalin, and embedded in paraffin for histological and immunohistochemical examination, or stored in RNA later (Qiagen) prior to RNA isolation, or subjected to flow-cytometric analysis.

### Fetal intestinal epithelial cell transplantation and treatment

Normal colon was obtained from 2 fetuses (#15 and #54, 21 and 23 weeks, respectively) from elective abortions performed at Magee Women's Hospital, Pittsburgh, PA. The colon was cut longitudinally in HBSS, contents rinsed, cut into 1-inch pieces, transferred to EBSS/10mM EGTA/1% HEPES (Life Technologies/Sigma-Aldrich/Mediatech) and minced. Tissue was then transferred to a tube and incubated for 5 min at room temperature. After an EBSS wash, the tissue was treated three times with a cocktail containing 1mg/mL collagenase II (Life Technologies), 1mg/mL hyaluronidase (Sigma-Aldrich), and 20μg/mL DNase I (Roche) in HBSS/1% HEPES for 20 min. Tissue/cell suspensions were passed through a 100m cell strainer to isolate single cells from undigested tissue.

For lymph node transplantation, recipient mice (BALB/c nude, n=12) were anesthetized with 1–3% isoflurane. A small incision was made in the abdomen to expose jejunal lymph nodes. A 25μL gas-tight removable needle syringe (Hamilton, 7656-01) with a removable needle (gauge 27) (Hamilton, 7803-01) was used to slowly inject the cell suspension (2×10^5^ cells/mouse) into a single lymph node. Light cauterization was used to seal the opening. The wound was then closed with surgical sutures. Ketoprofen (2 mg/kg, IM) treatment for postoperative pain relief was initiated right after surgery and continued for 2 additional consecutive days. Three weeks later, mice were divided in two groups; one group received i.p. injection of 20mg/Kg Torin-1 once a day for 12 days (n=7); the other group received vehicle (n=5). At the end of Torin-1 treatment, all mice were euthanized, their lymph nodes were collected, fixed 2 hours in 4% PFA, and embedded in paraffin for analysis. Sections were stained with H&E or Muc2 antibody (antibody used is indicated in Supplementary Table 1).

### Statistical Analysis

Data are presented as means ±SD. Statistical analysis was performed using Student's t test (p<0.05 was considered significant).

## Supplementary Methods, Figures and Tables




