# Supplementary material for: Selective targeting of human colon cancer stem-like cells by the mTOR inhibitor Torin-1
Source: Oncotarget. 2013 Sep 19;4(11):1948–62. doi: 10.18632/oncotarget.1310 (PMC3875761; doi:10.18632/oncotarget.1310)
Supplement: Supplementary file 1 [file oncotarget-04-1948-s001.pdf]

Selective targeting of human colon cancer stem-like cells by the mTOR inhibitor Torin-1- Francipane et al

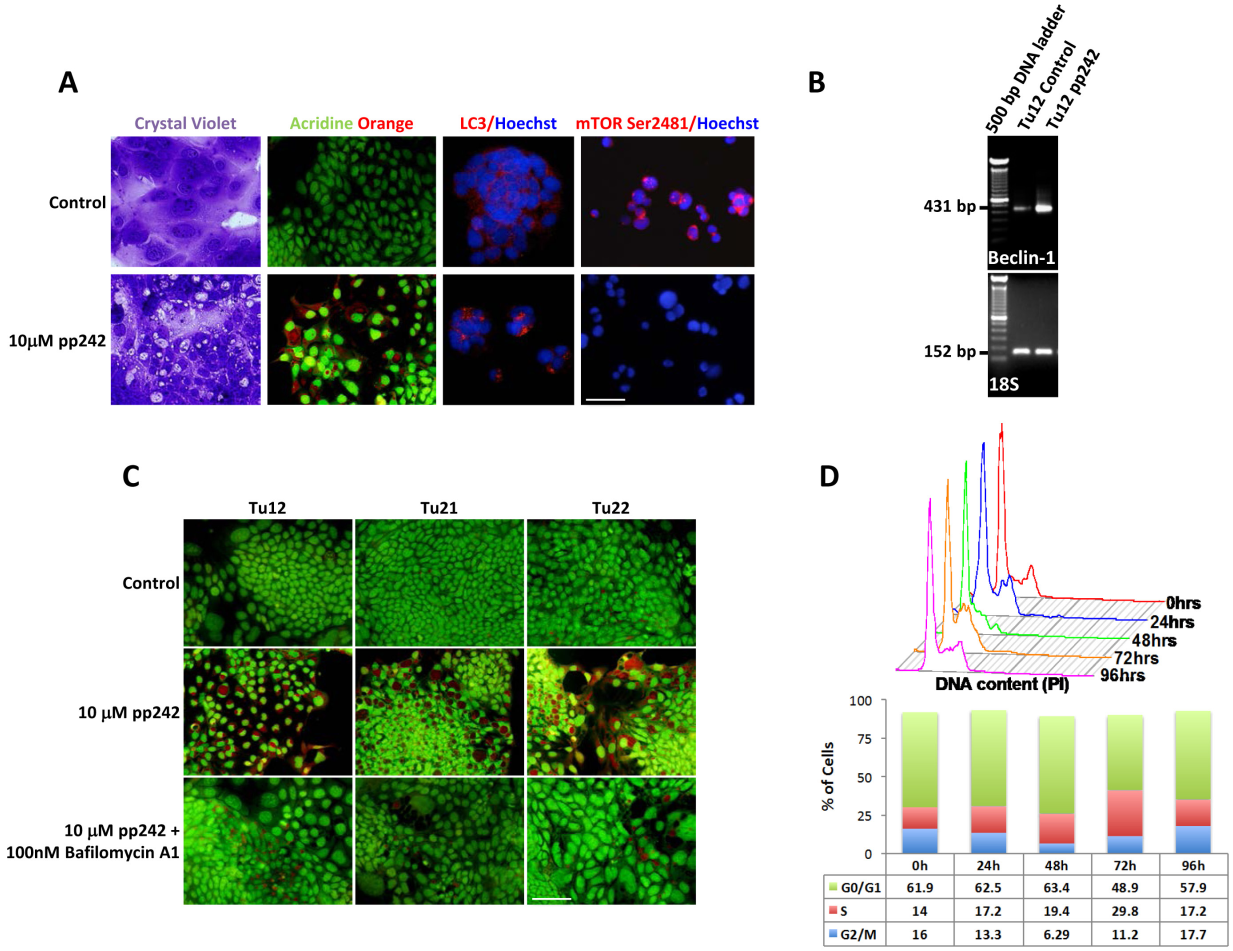

	0h	24h	48h	72h	96h
G0/G1	61.9	62.5	63.4	48.9	57.9
S	14	17.2	19.4	29.8	17.2
G2/M	16	13.3	6.29	11.2	17.7

Supplementary Figure 1: pp242 mTOR inhibitor induces protective autophagy in CoCSCs. (A) Pictures of CV-stain viewed by light microscopy, acridine orange fluorescent microscopy, or immunofluorescence of LC3 or mTOR Ser2481 in Tu12 cells treated with vehicle or 10µM pp242 for 48h. Scale bar, 100µm. Acridine orange-produced red fluorescence indicates AVO formation (adherent cells were incubated with 1µg/ml acridine orange for 15 min before taking pictures). (B) RT-PCR analysis for Beclin-1 or 18S housekeeping gene as internal control in Tu12 cells treated as in (A). (C) Pictures of acridine orange fluorescent microscopy of Tu12, Tu21, and Tu22 cells treated with vehicle, 10µM pp242 alone or in combination with 100nM Bafilomycin A1 for a total of 72 h (Bafilomycin A1 was added 48h after starting pp242 treatment. Scale bar, 100µm. (D) DNA content histograms (upper) and stacked bar graph (lower) showing the percentages of Tu12 cells in G0/G1, S, and G2/M phases of the cell cycle following treatment with 10µM pp242 up to 96h. Data from one of two representative experiments are shown. Cells were fixed with 70% ethanol, RNase treated (0.2mg/ml), and stained with PI (40µg/ml) before being analyzed with a flow cytometer. Data were re-analyzed with FlowJo software using the Dean-Jett-Fox curve fitting model.

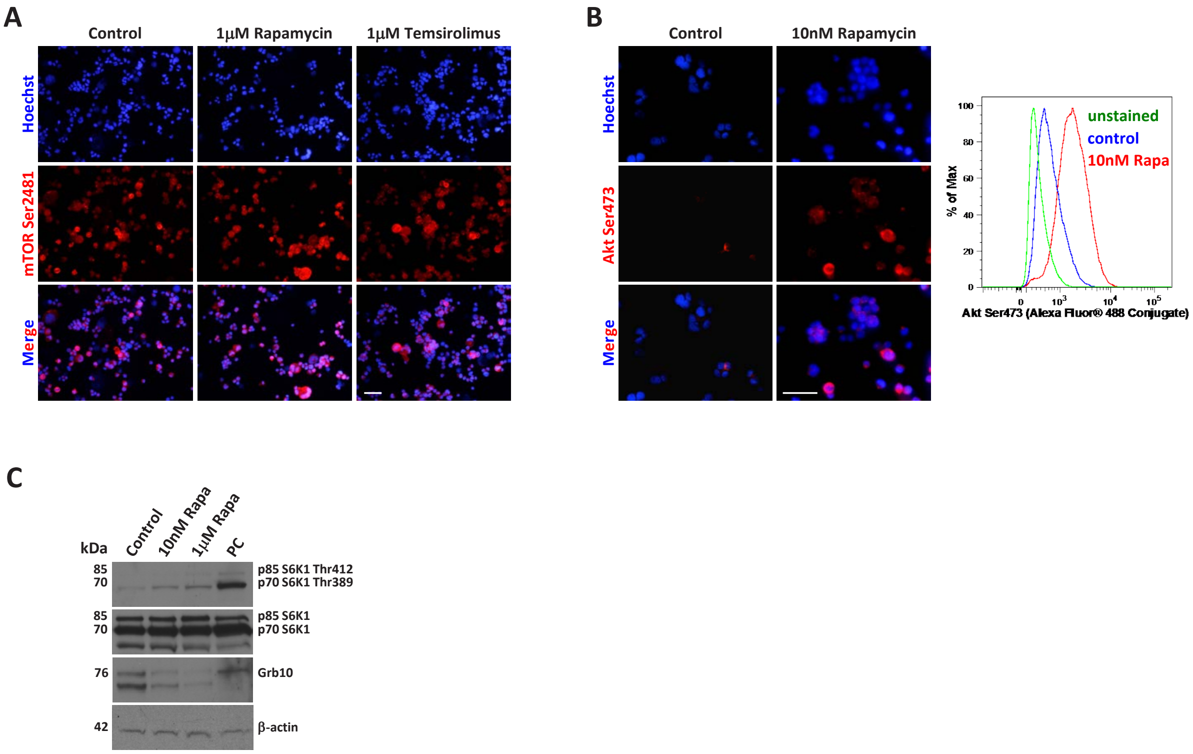

Supplementary Figure 2: First generation mTOR inhibitors fail to inhibit mTORC2 and lead to Akt activation in CoCSCs. Immunofluorescence pictures of Tu12 cells showing expression of (A) mTOR Ser2481 following incubation with Rapamycin or Temsirolimus (1μM, 48h) or vehicle as control, and (B, left panel) Akt Ser473 following incubation with Rapamycin (10nM, 48h) or vehicle as control. Scale bars, 100μm. (B, right panel) Flow cytometry histogram showing Akt Ser473 staining on PFA- and methanol-fixed Tu12 cells treated with Rapamycin (10nM, 48h) or vehicle as control. (C) Western blot images for phosphorylated or total p70 S6K1 Thr389/p85 S6K1 Thr412, Grb10 or β-actin as a loading control in Tu12 cells treated with 10nM or 1μM Rapamycin for 48h or vehicle as control. LA7 cells were used as positive control (PC).

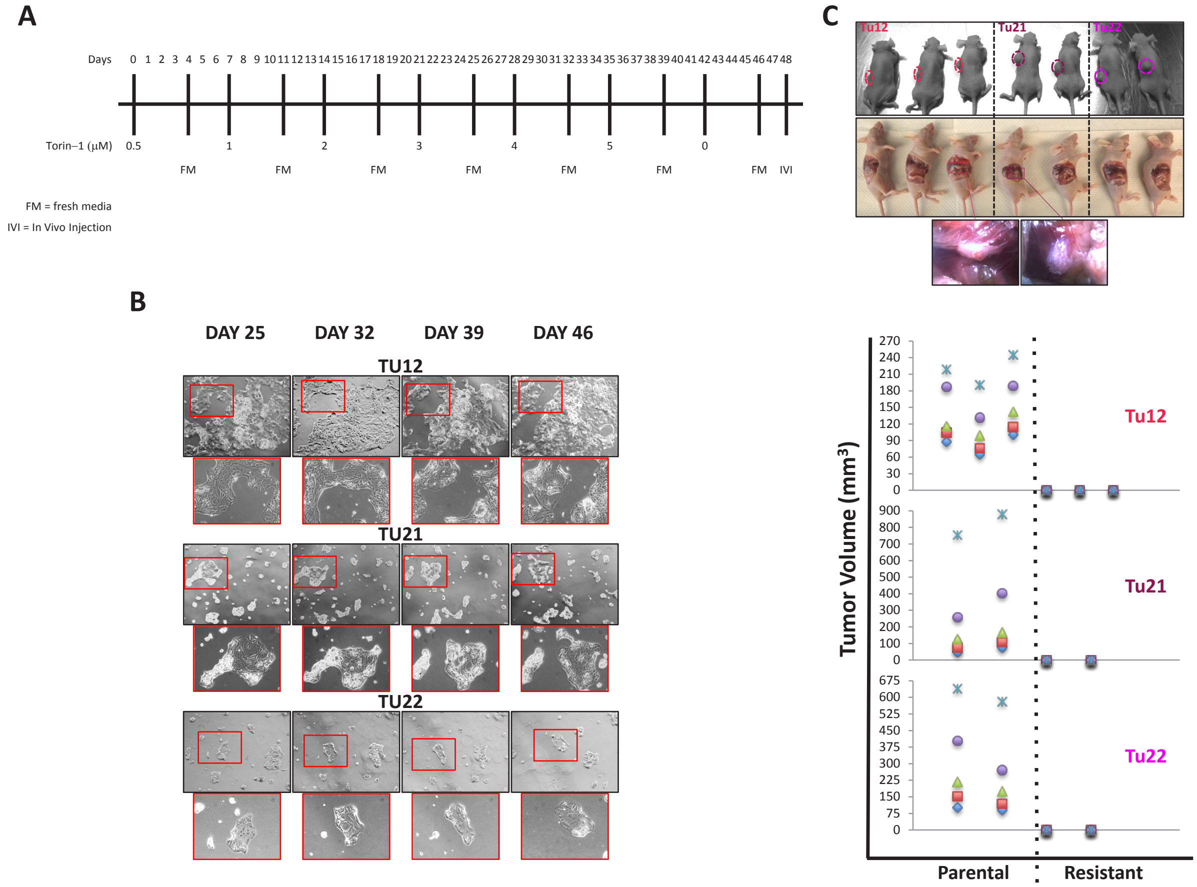

Supplementary Figure 3: Torin-1 resistant cells show a substantially reduced tumorigenic ability in vivo. (A) Overview of experimental plan: Torin-1 resistant cells were obtained by increasing concentrations of Torin-1 stepwise over a period of 7 weeks, starting at a concentration of 0.5µM up to a concentration of 5µM. Each dose was maintained in the medium for 96h after which cells were incubated in drug-free medium for additional 72h before being treated at the next higher drug dose. Following the 7-wk period both parental and Torin-1 resistant cells were injected s.c. into both flanks of nude mice (n=3 for Tu12; n=2 for Tu21 and Tu22). (B) Light microscopy pictures of resistant Tu12, Tu21, and Tu22 cells at day 25, 32, 39 and 46 showing low proliferation rate. (C, upper panel) Whole-body images of tumor-bearing mice at 7 weeks after tumor cell injection. Only parental cells gave rise to visible tumors (left flanks). The right flank of each mouse was skinned to expose tumors not visible to the naked eye. Two mice were found to have Torin-1 resistant cell-derived tumors. Stereoscope images of these tumors are shown. Dot plot graphs (lower panel) showing volumes (mm^3) of tumors generated by Tu12, Tu21, and Tu22 parental versus resistant cells up to 7 weeks.

Supplementary Methods

Reagents:

Antibodies used are indicated in Supplementary Table 1. Beclin-1 primers (sc-38913-PR) were purchased from Santa Cruz.
