# Supplementary material for: Selective targeting of human colon cancer stem-like cells by the mTOR inhibitor Torin-1
Source: Oncotarget. 2013 Sep 19;4(11):1948–62. doi: 10.18632/oncotarget.1310 (PMC3875761; doi:10.18632/oncotarget.1310)
Supplement: Supplementary file 2 [file oncotarget-04-1948-s002.docx]

| **Antibody** | **Cat No., Application(s)** | **Company** |
| --- | --- | --- |
| Akt Ser473 | 4058, IF | Cell Signaling Technology |
| Alexa Fluor® 488 and/or 594 secondary antibody conjugates | Flow | Invitrogen |
| Alexa Fluor® 488 Mouse anti-Akt (pS473) | 560404, Flow | BD Biosciences |
| Annexin V-FITC | 130-093-060, Flow | Miltenyi Biotec |
| APC CD29 | 559883, Flow | BD Pharmingen |
| APC CD326 | 130-091-254, Flow | Miltenyi Biotec |
| β-actin | Ab-1, WB | Calbiochem |
| Biotinylated CD44 | 555477, Flow | BD Pharmingen |
| Biotinylated MHC Class I | 121-030, Flow | Ancell |
| BV605 CD45 | 563053, Flow | BD Biosciences |
| CD31 | sc-1506, IF | Santa Cruz |
| CD44 | ab16728, IF | Abcam |
| Cleaved Caspase-3 Asp175 | 9664, IF, Flow | Cell Signaling Technology |
| FITC CD24 | 555427, Flow | BD Pharmingen |
| γ-Tubulin | sc-7396, IF | Santa Cruz |
| Grb10 | sc-74509, WB | Santa Cruz |
| LC3B | 2775, IF | Cell Signaling Technology |
| Lyve-1 | sc-80170, IF | Santa Cruz |
| mTOR | 2983, IHC | Cell Signaling Technology |
| mTOR Ser2448 | 5536, IF | Cell Signaling Technology |
| mTOR Ser2448 | 2976, IHC | Cell Signaling Technology |
| mTOR Ser2481 | ab45996, IF, IHC | Abcam |
| Muc2 | sc-7314, IHC | Santa Cruz |
| p70 S6 Kinase | 2708, WB | Cell Signaling Technology |
| p70S6K1 Thr389 | sc-11759, IF | Santa Cruz |
| PE CD166 | 559263, Flow | BD Pharmingen |
| PE CD49f | 555736, Flow | BD Pharmingen |
| PerCP/Cy5.5 CD326 | 2221070, Flow | iCyt |
| Phospho-p70 S6 Kinase Thr389 | 9234, WB | Cell Signaling Technology |
| PKCα Ser657 | sc-12356, IF, IHC | Abcam |
| Podoplanin | 11-009, IF | AngioBio |
| Pure MHC Class I | 121-20, IF | Ancell |
| SGK1 Ser422 | ab55281, IF, IHC | Abcam |
| Streptavidin APC | 554067, Flow | BD Pharmingen |
| Streptavidin APC Cy7 | 554063, Flow | BD Pharmingen |

**List of antibodies used**

| Gene | **Forward primer** | **Reverse primer** |
| --- | --- | --- |
| 18S | GTAACCCGTTGAACCCCATT | CCATCCAATCGGTAGTAGCG |
| Ki67 | CCCGTGGGAGACGTGGTA | TTCCCGTGACGCTTCCA |
| Muc2 | TGGCTGGATTCTGGAAAACC | TGGCTCTGCAAGAGATGTTAGC |
| Villin | AGCCAAGCAGTACCCACCAA | CTGAAAGACGGCCGACTCA |
| DLL1 | GGGCACCTTCTCTCTGATTATTGA | GGTTTTCTGTTGCGAGGTCATC |
| DLL4 | GGAGCTCAGCGAGTGTGACA | GCCATCCTCCTGGTCCTTACA |
| Notch1 | CATGGTACCAATCATGAATCTTTGTT | TCTGGAGGGACCAAGAACTTGT |
| Hes1 | ACTCCCCAACCCACCTCTCT | TCTTCTCTCCCAGTATTCAAGTTCCT |
| Bmi1 | GCAGCTCATCCTTCTGCTGAT | CATCTGCAACCTCTCCTCTATCTTC |
| Lgr5 | CCTGCGTCTGGATGCTAACC | GGAATGCAGGCCACTGAAA |
| CD44 | CAGATGGCATGAGGGATATCG | CTGCAGCTGTCCCTGTTGTC |
| GAPDH | GGCATCCTGGGCTACACTGA | GGAGTGGGTGTCGCTGTTG |

**Primers were designed using Primer Express software (Applied Biosystems)**
